# Oncovex MGM-CSF –mediated regression of contralateral (non-injected) tumors in the A20 murine lymphoma model does not involve direct viral oncolysis

**DOI:** 10.1186/2051-1426-3-S2-P336

**Published:** 2015-11-04

**Authors:** Keegan Cooke, James Rottman, Jinghui Zhan, Petia Mitchell, Oluwatayo Ikotun, Brittany Yerby, Angela Chong, Charles Glaus, Achim K Moesta, Beltran Pedro

**Affiliations:** 1 Amgen, Thousand Oaks, CA, USA; 2 Amgen, Boston, MA, USA; 3 Amgen, San Francisco, CA, USA; 4 Amgen Inc., South San Francisco, CA, USA; 5 Amgen Inc., Thousand Oaks, CA, USA

## Background

Talimogene laherparepvec (T-VEC) is an injectable modified oncolytic herpes simplex virus type-1 (HSV-1) hypothesized to be efficacious by at least two complimentary mechanisms of action: a) direct oncolysis of the injected tumor and b) elicitation of a systemic anti-tumor immune response against non-injected lesions and metastases. The purpose of this study was to test that hypothesis that direct viral oncolysis was not involved in the regression of contralateral (non-injected) tumors.

## Methods

A20 cells (2x10^6^) were injected subcutaneously on the right (injected) and left (contralateral) flank of female BALB/c mice. On day 10, mice were randomized into 4 groups based on tumor volume (~150 mm^3^, n=10/group). Tumor growth and body weight were measured twice per week throughout the experiment using calipers and an analytical scale, respectively. OncoVEX^mGM-CSF^ (T-VEC with murine GM-CSF), 3x10^4^-3x10^6^PFU/mouse, was delivered intratumorally every 3 days during the first week. Viral detection was performed in injected and contralateral tumors after a single injection of OncoVEX^mGM-CSF^. Four approaches were used to detect the presence of virus in contralateral tumors and tissues: ddPCR (viral DNA), Fluidigm analysis (viral gene mRNA), immunohistochemistry (HSV-1 protein) and PET imaging with [^18^F]FHBG (active thymidine kinase).

## Results

OncoVEX^mGM-CSF^ treatment caused tumor regression and complete cures in 10/10 injected tumors and 5/10 contralateral tumors when dosed intratumorally at 3x10^6^ PFU/mouse. Viral DNA was detected by ddPCR in all injected tumors, dose proportionally (4 mice per viral concentration, 5x10^3^, 5x10^4^, 5x10^5^ and 5x10^6^ PFU, total 16 mice). In contrast, viral DNA was only detected in 1/16 contralateral tumors (1 tumor in the 5x10^5^ PFU group). The level of viral DNA in this contralateral tumor was 1:1000 that detected in injected tumors and equivalent to that found in the blood of one mouse in the 5x10^6^ PFU group. HSV-1 viral gene expression analyzed by Fluidigm, viral capsid protein detected by immunohistochemistry, and HSV-1 thymidine kinase activity measured with [^18^F]FHBG PET were detected in all OncoVEX^mGM-CSF^ injected tumors between 24-168 hours post-infection. In contrast, no HSV-1 mRNA, protein, or thymidine kinase activity could be detected in contralateral tumors up to 168 hours post infection.

## Conclusion

The data presented here strongly suggests that direct viral oncolysis is not responsible for regression of contralateral tumors in the A20 murine lymphoma model. Detailed analysis of the adaptive immune response driven by OncoVEX^mGM-CSF^ in contralateral A20 tumors is currently being studied.

